# Neutralizing Concentrations of Anti-Botulinum Toxin Antibodies Positively Correlate with Mouse Neutralization Assay Results in a Guinea Pig Model

**DOI:** 10.3390/toxins13090671

**Published:** 2021-09-21

**Authors:** Milan T. Tomic, Shauna Farr-Jones, Emily S. Syar, Nancy Niemuth, Dean Kobs, Michael J. Hackett, Yero Espinoza, Zacchary Martinez, Khanh Pham, Doris M. Snow, James D. Marks, Ronald R. Cobb

**Affiliations:** 1 National Resilience, Inc., 2061 Challenger Dr., Alameda, CA 94501, USA; yero.espinoza@resilience.com (Y.E.); zacchary.martinez@resilience.com (Z.M.); Khanh.pham@alector.com (K.P.); 2 Department of Anesthesia and Perioperative Care, University of California, 1001 Potrero Ave., San Francisco, CA 94110, USA; shauna.farr-jones@ucsf.edu (S.F.-J.); jim.marks@ucsf.edu (J.D.M.); 3 Battelle Biomedical Research Center, West Jefferson, Columbus, OH 43162, USA; syare@battelle.org (E.S.S.); niemuth@battelle.org (N.N.); dkobs@amplify-bio.com (D.K.); mike.hackett@gmail.com (M.J.H.); 4 National Resilience, Inc., 13200 NW, Nano Ct, Alachua, FL 32615, USA; doris.snow@resilience.com (D.M.S.); RonCobb76@yahoo.com (R.R.C.)

**Keywords:** botulinum neurotoxin, botulism, aerosol, monoclonal antibody (mAb), guinea pig inhalation model, oligoclonal antibody, mouse neutralization assay (MNA), neutralizing antibody concentration (NAC)

## Abstract

Botulinum neurotoxins (BoNT) are some of the most toxic proteins known and can induce respiratory failure requiring long-term intensive care. Treatment of botulism includes the administration of antitoxins. Monoclonal antibodies (mAbs) hold considerable promise as BoNT therapeutics and prophylactics, due to their potency and safety. A three-mAb combination has been developed that specifically neutralizes BoNT serotype A (BoNT/A), and a separate three mAb combination has been developed that specifically neutralizes BoNT serotype B (BoNT/B). A six mAb cocktail, designated G03-52-01, has been developed that combines the anti-BoNT/A and anti-BoNT/B mAbs. The pharmacokinetics and neutralizing antibody concentration (NAC) of G03-52-01 has been determined in guinea pigs, and these parameters were correlated with protection against an inhalation challenge of BoNT/A1 or BoNT/B1. Previously, it was shown that each antibody demonstrated a dose-dependent mAb serum concentration and reached maximum circulating concentrations within 48 h after intramuscular (IM) or intraperitoneal (IP) injection and that a single IM injection of G03-52-01 administered 48 h pre-exposure protected guinea pigs against an inhalation challenge of up to 93 LD_50_s of BoNT/A1 and 116 LD_50_s of BoNT/B1. The data presented here advance our understanding of the relationship of the neutralizing NAC to the measured circulating antibody concentration and provide additional support that a single IM or intravenous (IV) administration of G03-52-01 will provide pre-exposure prophylaxis against botulism from an aerosol exposure of BoNT/A and BoNT/B.

## 1. Introduction

Botulism can be fatal if untreated and is caused by exposure to any one of the highly toxic protein family known as botulinum neurotoxins (BoNTs) [1,2,3,4], which are the most potent of all known biological poisons [5,6]. At least seven BoNT serotypes (A-G) have been reported [7,8,9]. An eighth serotype, BoNT/H has been reported [10], though its existence as a separate serotype is not universally agreed upon [11]. Serotypes are defined immunologically by the inability of the IgG antibodies that neutralize one serotype to neutralize the other serotypes [12]. BoNT serotypes A, B, E, and F cause the disease botulism in humans [13,14,15]. The majority of cases within the US are caused by BoNT/A and B, while worldwide, most botulism cases are caused by BoNT/A, B, and E, with BoNT/F being associated with a low incidence of food poisoning-related cases of botulism intoxication [1,2,3,4,13].

Due to the potential for use of BoNT by those of ill intent, the US Department of Defense has funded development of vaccines for BoNT/A and BoNT/B to protect warfighters from these serotypes [16]. An alternative to vaccination is prophylactic administration of safe recombinant human antibodies that neutralize BoNT [17,18,19,20,21,22]. Advantages of this passive immunization approach are that protection would be immediate and recipients would be able to receive therapeutic botulinum neurotoxin if needed subsequently [23].

The development of a potent human monoclonal antibody (mAb) based drug product, G03-52-01, composed of multiple mAbs binding to non-overlapping epitopes on BoNT/A and BoNT/B has been described previously [17,19,20,24,25,26]. The BoNT/A [27], BoNT/B [28], BoNT/E (NCT03603665, unpublished results), and BoNT/C/D [29] antitoxins have completed Phase 1 testing in humans, without serious adverse side effects.

Botulinum toxin exposure can occur through a variety of routes, but inhalation is considered the most likely route in bioterrorism or biowarfare settings [30]. Therefore, an inhalational model of toxin exposure using the guinea pig has been developed [31]. The recombinant BoNT/A and BoNT/B antitoxins have been shown to be efficacious in inhalation botulism models in guinea pigs [26].

The primary objective of this study was to investigate the pharmacokinetic (PK) profiles of G03-52-01 following intramuscular (IM) or intravenous (IV) administration to guinea pigs and to evaluate the correlation between circulating antibody concentrations and the neutralizing antibody concentrations (NAC), measured using a mouse neutralization assay (MNA). Several MNA measurements are required to obtain the average NAC for a given sample. The levels of circulating anti-BoNT/A mAbs were assessed using an enzyme-linked immunosorbent assay (ELISA) [25]. The levels of circulating anti-BoNT/B mAbs were assessed using an electrochemiluminescence (ECL) assay [24].

Establishment of a correlation between the NAC and MNA would reduce the need for conducting multiple MNA studies during the development of these antibody drugs. The National Institute of Health (NIH) has identified finding alternatives to the use of animals for the BoNT mouse bioassay as a priority. The Scientific Advisory Committee on Alternative Toxicological Methods, which advises the Interagency Coordinating Committee on the Validation of Alternative Methods (ICCVAM) and the National Toxicology Program Interagency Center for the Evaluation of Alternative Methods (NICEATM), also considered the development and validation of alternatives to the mouse LD_50_ assay for BoNT potency testing a high priority [32]. Using a large number of animals for the efficacy/toxicity testing of botulinum toxins is also at odds with the concepts of reduction, replacement, and refinement, adopted by the European Union and the Organization for Economic Co-operation and Development, which suggests the development of alternative test methods that do not require animals for such studies [33].

## 2. Results

### 2.1. Intravenous (IV) Administration of Anti-BoNT/A Antibodies

**Figure 1:**
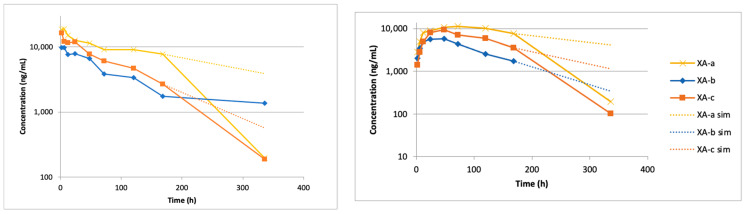
Concentration–time curves following IV (**left**) and IM (**right**) administration of 1.5 mg total antibody XA-a, XA-b, and XA-c; solid lines represent ELISA-measured concentrations and dashed lines represent predicted concentrations based on noncompartmental analysis of the data through 168 h.

All the IV administered antibodies in the anti-BoNT/A mAbs exhibited a biphasic curve consistent with 2-compartment kinetics through 168 h (7 days). The mAb concentration at the final timepoint at 336 h (14 days) was very low compared to the earlier timepoints for the XA-a and XA-c mAbs and did not fall on the biphasic curve (Figure 1); after 168 h there was a large increase in mAb clearance. As the kinetics of clearance change over time, the data could not be modeled compartmentally through 336 h. The XA-a and XA-c antibodies could not be detected after 336 h, suggesting an anti-drug antibody (ADA) response was likely generated against the mAbs. To fully characterize the PK properties, the data were modeled two ways: (1) the data through 168 h was fit to a 2-compartment model representing the intrinsic kinetics of the antibody without the putative ADA response, and (2) the data were fit to a non-compartmental model through 336 h. The latter method provides the most accurate representation of exposure in the animal.

**Figure 2:**
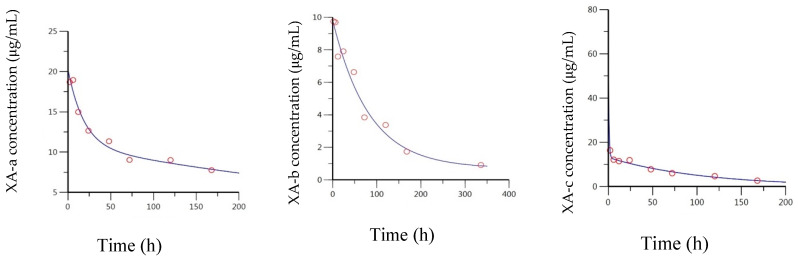
Concentration–time data for IV-administered XA-a, XA-b, and XA-c. Blue line indicates best line fit, based on a two-compartment model using Phoenix WinNonlin. A single point (t = 2 h) represents the distribution phase for XA-c, which biased the model to a large C_0_ of 66 μg/mL.

The measured versus predicted concentration–time curve for XA-a, XA-b, and XA-c from the 2-compartment model is presented in Figure 2, and the derived parameters are presented in Table 1. While all the data fit a two-compartment model better than a one-compartment model, there was substantial variability between the curves. XA-a had a well-characterized distribution and elimination phase, with equilibrium being established by approximately 50 h. XA-b had a much longer distribution phase, such that the first point on the terminal phase was at 168 h. A concentration estimate for XA-b at 336 h was used to define the terminal phase. XA-c had an extremely fast distribution phase, such that the second timepoint at 6 h appears to be on the terminal phase. This made it difficult to accurately model the distribution phase, which led to a notably high estimate of C_0_ (the concentration immediately upon injection). The elimination rate half-lives (t_½_ elim) estimated from this analysis were 201 h and 447 h, for XA-a and XA-b, respectively. Since the XA-c was cleared much faster, its estimated elimination rate half-life was 14.8 h. Despite the short elimination rate half-life, mAb concentrations could be measured through 336 h. This is because the mAb distributes between compartments at a slower rate, thus limiting clearance. Consequently, the beta-phase half-life for XA-c was 73.5 h. The beta-phase half-life is the slope of the terminal phase in a 2-compartmental model and is dependent on the rates of distribution between compartments, in addition to the elimination rate constant. The long half-lives resulted in large area under the curve (AUCs), of 5880, 6330, and 1430 h*μg/mL for XA-a, XA-b, and XA-c, respectively. These AUC estimates are the result of an extrapolation through infinite time with no ADA. Thus, they should be considered overestimates of the exposures in smaller animals but may be more useful in extrapolating exposures to humans, where an ADA response is less likely.

The full data set was analyzed using the non-compartmental model to provide a more accurate representation of the data that were measured, and the results are summarized in Table 2. The terminal phase half-life (i.e., the β-phase) was greatly decreased for XA-a and XA-c, due to the sharp reduction in concentrations at 336 h, with respective half-lives of 35.2 and 34.4 h. XA-b was less affected in the terminal phase estimation of 93.9 h, due to the lack of a measurable concentration at 336 h. AUC_last_ was used as the estimate of exposure for noncompartmental analysis, because the concentration dropped off rapidly for XA-a and XA-c, so there was no difference between AUC_last_ and AUC_inf._ The concentration at 336 h allowed for significantly increased AUC_inf_ for XA-b; without this data point the AUC_inf_ for XA-b would have been well over 20% extrapolated. Thus, the estimates for AUC_last_ are 2410, 1020, and 1390 h*μg/mL for XA-a, XA-b, and XA-c, respectively.

### 2.2. Pharmacokentics Following Intramuscular (IM) Administration of Anti-BoNT/A mAbs

**Figure 3:**
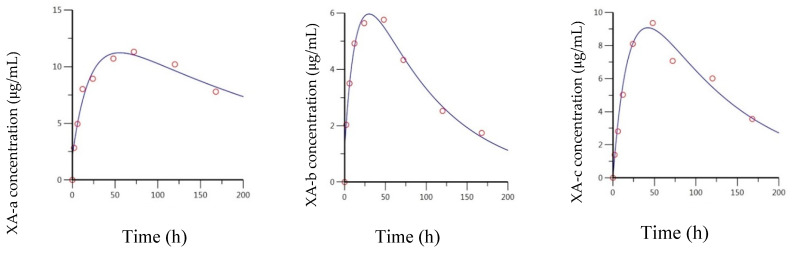
Concentration–time data for IM-administered XA-a, XA-b, and XA-c. Blue line indicates best fit, based on a one-compartment model using Phoenix WinNonlin.

When the anti-BoNT/A mAbs were administered IM, all three mAbs exhibited a slow absorption phase followed by the elimination phase. In the case of all three mAbs, a biphasic curve could not be seen after the C_max_, thus an extravascular one-compartment model provided a better fit for the data. Like the results observed with IV administration, all three of the mAbs could be modeled through 168 h. At the next timepoint, 336 h, XA-b was not measurable while XA-a and XA-c showed an expedited clearance (Figure 1). The measured versus predicted concentration–time data for XA-a, XA-b, and XA-c, based on the compartmental model are provided in Figure 3. Based on the model, the respective estimates of the absorption half-life for XA-a, XA-b, and XA-c, were 14.3, 10.2, and 13.8 h, which corresponded to T_max_ values (time for antibody for reach maximum level) of 55.4, 29.9, and 42.0 h. Estimates of the elimination half-life were 202, 64.1, and 78.2 h, respectively, for XA-a, XA-b, and XA-c. While the elimination rates for XA-a were very similar for both routes of administration, the elimination rate was lower for XA-b and greater for XA-c administered via IM, due to the limitations in fitting the models described above. Extrapolated estimates of AUC were determined to be 4000, 781, and 1480 h*μg/mL, respectively, for XA-a, XA-b, and XA-c.

Noncompartmental analysis of the IM concentration–time data resulted in terminal elimination half-life estimates of 24.5, 73, and 30.4 h for XA-a, XA-b, and XA-c, which agree with the compartmental estimates. However, the terminal elimination half-life of XA-a was underestimated substantially, due to the 336 h timepoint (Figure 3). Estimates for AUC_last_ were 2260, 627, and 1370 h*μg/mL for XA-a, XA-b, and XA-c, respectively. Since the IM dose was equivalent to the IV dose, the ratio is also a reflection of the absolute bioavailability, F. Based on these estimates, XA-a was 94% bioavailable, XA-b was 83% bioavailable, and XA-c was 99% bioavailable. The lower estimate for XA-b is due in part to the lack of a value at the 336 h timepoint in the concentration–time curve following IM administration. However, the concentrations at 168 h were similar following both IM and IV administration of XA-b, due to the longer terminal half-life estimated following IV administration; thus, even if the AUC is extrapolated, it will not increase the bioavailability greatly.

### 2.3. Pharmacokentics Following Intravenous (IV) Administration of Anti-BoNT/B mAbs

**Figure 4:**
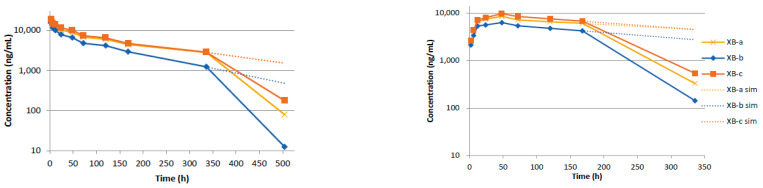
Concentration–time curves following IV (**left**) and IM (**right**) administration of 1.5 mg total antibody XB-a, XB-b, and XB-c; solid lines represent ECL-measured concentrations. Dashed lines represent predicted concentrations, based on noncompartmental analysis of the data through 336 h (IV) or 168 h (IM).

The anti-BoNT/B mAbs exhibited less variability between constituent antibodies compared to the anti-BoNT/A mAbs and were more amenable to modeling. A biphasic curve is observed for all three mAbs. Additionally, the concentration–time curves suggest similar distribution and elimination rates for all the antibodies, as all curves are parallel; XB-a and XB-c are nearly superimposable (Figure 4). The kinetics appear to diverge from the model after 336 h; however, the same phenomenon of rapidly decreasing concentrations is observed. The concentration–time data for the anti-BoNT/B mAbs were analyzed by fitting a two-compartment model through 336 h, to determine the intrinsic PK parameters. Estimates of exposure and bioavailability were derived based on a non-compartmental model.

**Figure 5:**
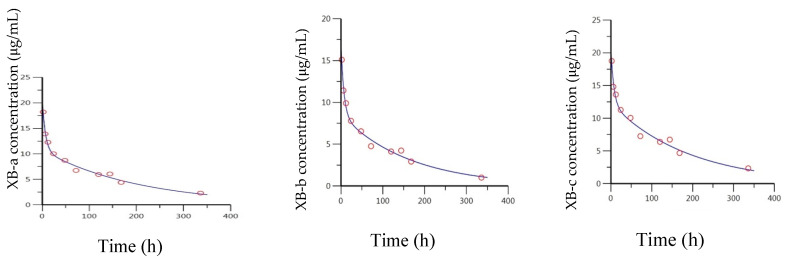
Predicted and observed concentration–time data for IV-administered XB-a, XB-b, and XB-c, based on a 2-compartment model fit by Phoenix WinNonlin.

The measured versus predicted concentration for all three anti BoNT/B mAbs is presented in Figure 5. All three models are nearly identical, with a clear distribution phase through 24 h, before the terminal phase becomes apparent. Based on the model, XB-b appears to have a slightly decreased C_0_ of 17.2 μg/mL, compared to 20.7 μg/mL for XB-a and XB-c. This is potentially due to a difference in dosing, as the entire XB-b curve is shifted slightly lower compared to the two other curves. The derived parameters from all three curves are in good agreement with each other (Table 2). Estimates of elimination half-life ranged from 59.4 to 81.1 h, β-phase half-lives ranged from 115 to 144 h, and AUC estimates ranged from 1470 to 2420 h*μg/mL for each of the XB antibodies.

Noncompartmental analysis of the concentration–time data provided similar estimates to the compartmental model for IV administration. Since so many timepoints were used in the estimation of the terminal phase, the significantly lower concentration at 504 h of the XB mAbs (Figure 4) did not greatly affect estimates. Terminal phase half-lives of 69.5, 49.6, and 77.2 h were estimated for XB-a, XB-b, and XB-c, respectively. Similar estimates of exposure were also determined compared to the compartmental data; 2060, 1380, and 2220 h*μg/mL for XB-a, XB-b, and XB-c, respectively.

### 2.4. Pharmacokentics Following Intramuscular (IM) Administration of XB mAbs

Following IM administration, the anti-BoNT/B mAbs were absorbed faster than the XA mAbs. Like the XA mAbs, after C_max_ a biphasic profile was not observed, thus a 1-compartment extravascular model fit the data better than a 2-compartment model. In addition, measurable concentrations were observed at 336 h for all three mAbs; however, the 168 h timepoint was again the inflection point for a change in clearance (Figure 4). Therefore, the data were modeled in the same manner as the anti-BoNT/A mAbs, with a compartmental model to estimate the intrinsic PK parameters and a noncompartmental analysis for measured values.

**Figure 6:**
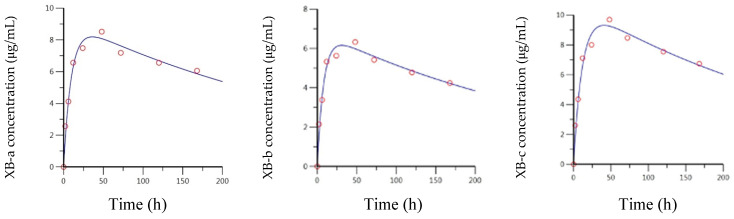
Predicted and observed concentration–time data for IM-administered XB-a, XB-b, and XB-c, based on a 2-compartment model fit by Phoenix WinNonlin.

The measured versus predicted concentrations for all three mAbs are presented in Figure 6, and a summary of the PK parameters is provided in Table 3 and Table 4. All three mAbs exhibited very similar absorption and elimination profiles; again, with the XB-a and XB-c profiles essentially superimposed and the XB-b profile shifted slightly downward. The anti-BoNT/B mAbs were absorbed, approximately, twice as fast as the anti-BoNT/A mAbs, with absorption half-lives of 6.71, 5.59, and 8.08 h, for XB-a, XB-b, and XB-c, respectively. The corresponding T_max_ estimates were 36.1, 31.0, and 40.7 h, respectively. Elimination half-lives were very consistent, with estimates of 254, 237, and 236 h, respectively, for XB-a, XB-b, and XB-c, as reflected in the essentially parallel curves of Figure 6. The resulting AUCs were also very similar; 3310, 2300, and 3570 h*μg/mL, for XB-a, XB-b, and XB-c, respectively.

Due to the sharp decrease in concentration for all three mAbs at 336 h, the estimated terminal phase half-lives were greatly reduced in the noncompartmental analysis. For XB-a, XB-b, and XB-c, estimates of 38.1, 26.5, and 46.3 h were calculated, which were approximately 5- to 8-fold lower than those estimated from the compartmental analysis. Corresponding AUC_last_ estimates were 1670, 1210, and 1890 h*μg/mL. When these estimates of exposure were compared to those for IV, bioavailability estimates of 81%, 88%, and 85% were calculated for XB-a, XB-b, and XB-c, respectively. These estimates are similar but slightly lower than those for the anti-BoNT/A mAbs.

### 2.5. Correlation of Pharmacokinetics and MNA for Anti-BoNT/A and /B mAbs

**Figure 7:**
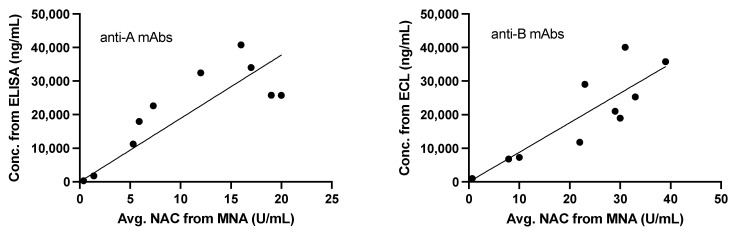
Comparison of anti-BoNT/A and /B mAbs average concentrations measured by ELISA or ECL and MNA. Measurements from animals dosed via IM and IV are combined for this analysis. The line was calculated from regression and included the 0, 0 datapoint. Regression and correlation parameters are shown in Table 5.

The concentration of the mAbs measured by ELISA or ECL was compared with that measured by MNA. Blood samples were collected at 6, 12, 24, 48, and 336 h (14 days) post-dosing, following either IV or IM administration. After anti-BoNT/A mAb IM administration, the NAC increased from 5.3 Units/mL at 6 h to 19 Units/mL at 48 h post-administration and then decreased to less than 0.14 Units/mL at 336 h (Figure 7). The NAC 6 h after anti-BoNT/A mAb IV administration was 16 Units/mL and remained elevated at 12 h, 24 h, and 48 h, with values of 17, 12, and 20 Units/mL, respectively, before decreasing to 1.4 Units/mL at 336 h. For anti-BoNT/B mAbs administered IM, the average NAC increased, from 10 Units/mL at 6 h to 30 U/mL at 24 h. The NAC remained at 29 Units/mL at 48 h and decreased to less than 0.45 Units/mL at 336 h. The anti-BoNT/B mAbs administered IV had a NAC of 31 Units/mL at 6 h post-administration. Overall, the NAC per group remained elevated at 12, 24, and 48 h, with values of 39, 23, and 33 Units/mL, respectively. The NAC decreased to less than 5.3 Units/mL at 336 h.

**Figure 8:**
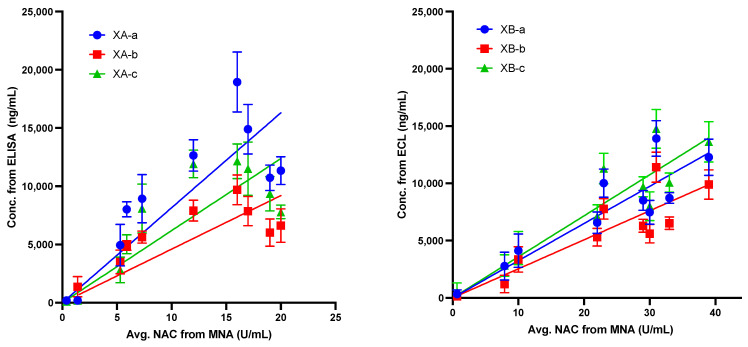
Comparison of individual anti-BoNT/A and B mAbs concentrations measured by ELISA or ECL and average (avg.) neutralizing antibody concentration (NAC) from the mouse neutralization assay (MNA). Measurements from animals dosed via IM and IV are combined for this analysis. The line was calculated from regression and included the 0, 0 datapoint. Regression and correlation parameters are shown in Table 5. Error bars indicate standard deviation. Linear regression and correlation were calculated with Prism v9.0. 0, 0 data point included.

Since the serum drug concentration is independent of the route of administration, the antibody concentrations at each timepoint were compared to the average NAC from both IV and IM assays. Total antibody concentrations (XA-a, XA-b, and XA-c, or XB-a, XB-b, and XB-c) at each timepoint, as determined using ELISA or ECL, respectively, were compared to the average NAC. The NAC values and mAb concentration values were then plotted. The individual mAb concentrations correlated well with the average NAC, indicating a linear correlation between circulating antibody and the protection observed, as measured by the NAC (Figure 8). For all mAbs, the slope was significantly different from zero, *p* < 0.0001 and the correlations were significant (alpha = 0.05). Parameters from linear regression analyses are shown in Table 5. The R^2^ for XA-a was 0.6934, for XA-b was 0.5765, and for XA-c was 0.6795. The correlation between average NAC and MNA was stronger for the anti-B mAbs. The R^2^ for XB-a was 0.8012, for XB-b was 0.7659, and for XB-c was 0.821.

The average NAC is a result of the three circulating antibodies, due to the mechanism of action [17], thus the concentrations of the three anti-BoNT/A and anti-BoNT/B were averaged and plotted against the average NAC to determine the correlation (Figure 7). As the average NAC increases, the molecular concentration increases, establishing a significant correlation between the circulating antibody concentration and protection, indicated by the average NAC observed. For both anti-A and anti-B mAbs, the slope is significantly different from zero, *p* < 0.0001 and the correlations are significant (alpha = 0.05).

It was previously shown that the concentration of each of the six antibodies that comprise G03-52-01 demonstrate peak or near-peak mAb serum concentrations 48 h after IM injection in guinea pigs [26] and that the antibody concentrations of each of the antibodies could be detected up to 28 days post-IM injection. Here, we found NAC up to 14 days post-IM injections, with doses of 1.5 mg/animal for anti-BoNT/A and 1.5 mg of anti-BoNT/B mAbs, which are correlated with the efficacy and mouse NAC determinations.

## 3. Discussion

Botulism is a neuroparalytic syndrome that can progress to respiratory failure and death without rapid treatment [34]. Passive immunization against botulism can be achieved by administering neutralizing antibodies [35,36,37]. The only FDA approved therapies, polyclonal antibodies such as botulinum immune globulin (BIG-IV) [38] and BAT^®^ [39], have severe limitations [40,41] that can be overcome by recombinant mAbs.

MNA is the gold standard method for determining the potency of an antitoxin and is used to demonstrate the NAC for a given sample [42]. However, the mouse bioassay requires hundreds of animals a week to perform, is expensive, and is subject to the significant variability inherent in a bioassay. Thus, a more rapid in vitro method to determine the potency of antitoxin mAbs would be a considerable advance in the development of these antibody drugs.

Here, we correlated the MNA and the average NAC measured with the circulating antibody concentration for anti-BoNT/A and anti-BoNT/B mAbs delivered via IM and IV. Comparison of the NAC and measured circulating antibody concentration allows for the establishment of a correlation between the presence of antibody in the sera and the NAC. Circulating antibodies, as determined by ELISA or ECL, and neutralizing antibodies, as determined by the MNA, reached a peak concentration in guinea pigs within 48 h post-administration, and the correlation with the average NAC measured demonstrates the causative effect of the antibodies in circulation. This correlation provides evidence in support of the use of circulating antibody concentration as measured by ECL or ELISA as a replacement for the MNA.

All the anti-BoNT/B antibodies exhibited a dramatic change in kinetics between 336 and 504 h (Figure 4), when the long terminal half-lives that are characteristic of mAbs abruptly decreased. The timing and effect on the concentration–time profile is consistent with an ADA response that occurred at approximately the same time and was independent of the route of administration. While we did not measure ADA in the guinea pig, we did measure ADA in rat [43]. There is no expectation that guinea pigs would be unique in not generating ADA. The effect of ADA would be to reduce the drug antibodies from circulation and because they would not bind to toxin, they would not interfere with the ECL or ELISA. From the PK analysis, the anti-BoNT/A mAbs exhibited a greater physiological variability than the anti-BoNT/B mAbs. Each of the six IgG1 antibodies studied here has the same constant region, with different variable regions. The reason for the variation in PK for each antibody is unknown. This can be seen in the widely variable distribution phases following IV administration, as shown in Figure 2. The same was observed following IM injection of the anti-BoNT/A mAbs (Figure 3). The absorption and elimination profiles are variable for each antibody, resulting in three dissimilar curves. Due to this, the exposure to each antibody was highly variable: 1020–2410 h*μg/mL (IV, non-compartmental analysis), 1430–6330 h*μg/mL (IV, compartmental analysis), 627–2260 h*μg/mL (IM, non-compartmental analysis), and 781–4000 h*μg/mL (IM, compartmental analysis). The concentration–time profiles for anti-BoNT/B mAbs, on the other hand, were nearly superimposable following both IV and IM administration (Figure 4). The XB-b profile was consistently below the other two curves, but resulted in similar C_max_ and AUC estimates, independently of the route of administration or method of analysis.

## 4. Conclusions

This work continues previous efficacy investigations with three antibody combinations to BoNT serotypes A and B, which were shown to completely protect guinea pigs against lethal aerosolized doses of BoNT/A and BoNT/B [26]. To protect against BoNT/A and BoNT/B intoxication, a single cocktail of antibodies has been co-formulated in a lyophilized presentation, delivered IM for ease and speed of delivery.

The statistically significant correlation of the circulating antibody concentration with the serum NAC has been demonstrated in this study. Limitations of the MNA for PK modeling include intrinsic variability of the assay, high cost, long assay time, and the requirement for large numbers of animals. This study provides evidence to support the use of the measurement of the circulating antibody concentration as a surrogate for the MNA.

## 5. Materials and Methods

### 5.1. Antibodies

An oligoclonal mixture of six IgG monoclonal antibodies (mAbs) against BoNT/A and BoNT/B, G03-52-01, comprised of a lyophilized, equimolar mixture of mAbs XA-a, XA-b, XA-c, and mAbs XB-a, XB-b, XB-c [24,25,26,27,28,44].

### 5.2. Animals and Animal Welfare

For the PK studies, a total of 108 male and female guinea pigs *(Cavia porcellus*, Crl: [HA]Br) purchased from the Charles River Laboratory were used. Guinea pigs were individually housed, with ad lib PMI, Inc., guinea pig chow and water, as previously described [36], except that a 12 h light–dark cycle was used. At the end of the study guinea pigs were euthanized by CO_2_ gas.

For the MNA studies, 240 male CD-1 (ICR) mice (*Mus musculus*) purchased from the Charles River Laboratory and weighing 17 to 23 g were used. Mice were individually housed with ad lib PMI, Inc. rodent chow and water under the same conditions as the guinea pigs. Mice that survived the 96 ± 2-h post-challenge observation period were euthanized by CO_2_ gas.

The research was conducted in compliance with the Animal Welfare Act (AWA, 7 U.S.C. §2131, 2002, 2007, and 2008) and other federal statutes and regulations relating to animals and experiments involving animals and adhered to the principles stated in the Guide for the Care and Use of Laboratory Animals (Battelle Biomedical Research Center, Columbus, OH, USA, Protocol Number 91734, approved 6 December 2017). Battelle Biomedical Research Center provides the only FDA approved, statistically validated MNA for determining anti-BoNT antibody concentrations (NACs) in the United States [16]. All animal procedures were conducted under protocols approved by the Institutional Animal Care and Use Committees (IACUC) of Battelle Biomedical Research Center, in accordance with IACUC guidelines [45]. General procedures for animal care and housing were in accordance with the Association for Assessment and Accreditation of Laboratory Animal Care International (AAALAC) recommendations.

### 5.3. Pharmacokinetics (PK)

PK profiles of circulating anti-BoNT/A antibodies were assessed using an enzyme-linked immunosorbent assay (ELISA). The levels of circulating Anti-BoNT/B antibodies were assessed using an electrochemiluminescence (ECL) assay, as described [26]. Guinea pigs *(Cavia porcellus*, *Crl: [HA]Br)* were studied in three cohorts, PBS, G03-52-01 at doses of 3 mg/animal delivered by IM or IV. A total of 108 male and female guinea pigs were used (12 controls, 48 each for IV and IM administration of G03-52-01). Samples were analyzed for PK with the ECL or ELISA assay at timepoints of 2 h, 6 h, 12 h, 24 h, 48 h, 72 h, 120 h, 168 h, 336 h, 504 h, 672 h, 840 h, 1008 h, 1344 h, and 1680 h. Due to the large volume of samples required to perform the MNA, samples were analyzed at timepoints of 0, 6, 12, 24, 48 h, and 14-day post-antibody dose using the MNA. The amount of toxin neutralization afforded by the antibodies NAC was assessed using the MNA with BoNT/A1 and BoNT/B1, with concomitant ECL/ELISA mAb concentration measurements for the PK.

### 5.4. Electrochemiluminescence (ECL) Assay for Anti-BoNT/B mAbs

The levels of circulating Anti-BoNT/B antibodies, XB-a, XB-b, and XB-c, were assessed using an electrochemiluminescence (ECL) assay. The ECL method to measure mAb concentration in guinea pig serum is based on a bridging immunoassay using the Meso Scale Discovery (MSD) electrochemiluminescence (ECL) format [26,27,44]. The antibody specific domains used are recombinant domains of BoNT/A or /B [24,25]. Biotinylated and ruthenylated domains were used as the capturing and detecting reagents for the assay, respectively. The assay uses the bivalent binding capability of the antibodies to form a bridging complex with biotinylated domain and ruthenylated domain to generate ECL signals for the measurement of the target antibody concentration in serum.

Briefly, a solution of reaction mixture containing both biotinylated domain and ruthenylated domain plus either calibration standards, assay acceptance controls, or study samples was incubated for 1 h at room temperature on an orbital shaker and shielded from light. The calibration standards and assay acceptance controls were prepared using drug products G03-52-01 containing six mAbs XA-a, XA-b, XA-c, XB-a, XB-b, and XB-c [24,25]. The standards were prepared by spiking G03-52-01 into neat guinea pig serum to make the highest concentration standard sample at 500 ng/mL (1 X assay concentration). The subsequent standard points were prepared as described [26].

Three separate assay methods were used to measure concentrations of the anti-BoNT/B mAbs, XB-a, XB-b, and XB-c in guinea pig serum. In each method standards, controls, and samples were diluted at the minimum required dilution of 1:20, with some samples being further diluted into the standard curve range. Each diluted standard, control, and sample was then combined with a solution containing the biotin labeled and ruthenium labeled forms of one of three recombinant domains of BoNT/B in a 96-well polypropylene plate and incubated for 12 to 18 h at room temperature on a shaker shielded from light. Once this incubation had been completed samples were transferred from the polypropylene plate to a streptavidin plate blocked with Blocking Buffer and incubated for an additional 2 h at room temperature on the shaker. The plate was washed and 2X read buffer was added to the plate before reading on the MSD Meso QuickPlex SQ 120.

The MSD instrument detected the chemiluminescent signal generated when an electric current was applied. The resulting signal was measured in ECL units. The calibration curve was plotted using a 4-parameter logistic curve fit using 1/y^2^ weighting. The concentrations of each analyte in the quality control (QC) and study samples were interpolated from the calibration curve generated.

### 5.5. ELISA Assay for Anti-BoNT/A mAbs

Three separate enzyme-linked immunosorbent assay (ELISA) methods were used to measure concentrations of the BoNT/A mAbs, XA-a, XA-b, and XA-c in guinea pig serum, as previously described [25] with modifications. In the XA-a, XA-b, and XA-c assay, the domains were coated at concentrations of 1.0 µg/mL, 20.0 µg/mL, and 5.0 µg/mL; these are recombinant domains of botulinum toxin A. Nunc Maxisorp 96-well plates are used in each assay. After a coat incubation period of 12–24 h, each plate was washed and blocked for 1–4 h. Standards, controls, and samples were diluted at the minimum required dilution of 1:20, with some samples being further diluted into the standard curve range. Diluted standards, controls, and samples were added to the coated and blocked plate for three hours at room temperature on a shaker. During sample incubation, the XA-a, XA-b, and XA-c antibodies in guinea pig serum bind with the coated domains. The plate was then washed and goat anti-human IgG (H + L)-HRP was added to the plate and incubated for an additional hour. The plate was washed and 3,3′,5,5′-Tetramethylbenzidine (TMB) substrate solution was added to the plate and incubated in the dark for approximately 20 min before the addition of stop solution. The plate was read on a microplate reader at 450 nm for detection and 630 nm for reference.

A calibration curve was generated from the resulting absorbance values and plotted using a 4-parameter logistic curve fit using 1/y^2^ weighting. The concentrations of each analyte in the QCs and study samples were interpolated from the calibration curve generated.

### 5.6. Measurement of Neutralizing Antibody Concentration (NAC) and Mouse Neutralization Assay (MNA)

NACs were determined using a standardized and statistically-validated MNA based on methods developed by Cardella and Hatheway and Dang [46,47], as described previously [26]. Male CD-1 (ICR) mice (*Mus musculus*) purchased from the Charles River Laboratory and weighing 17 to 23 g were used for the MNA. A total of 240 mice were used (five timepoints x ten mice per group x two serotypes x two routes of administration, plus controls). The BoNT challenge materials were dilutions of the complex form of BoNT/A subtype A1 and BoNT/B subtype B1 purchased from Metabiologics (Madison, WI, USA). The BoNT/A1 was produced from a *C. botulinum* Hall A strain. The BoNT/B1 was produced from the *C. botulinum* Okra strain. BoNT and samples for the mouse neutralization assays (MNA) were diluted in 30 mM phosphate buffered saline (PBS, pH 6.2) containing 0.2% (*w*/*v*) gelatin. Eight, four-fold dilutions were prepared for each serum sample. The antitoxin reference standards and guinea pig serum samples were titrated against a fixed amount of BoNT (31 mouse LD_50_/mL for BoNT/A1 and 19 mouse LD_50_/mL for BoNT/B1), incubated for 60 to 120 min at room temperature and 0.2 mL was injected IP into mice. The NAC was calculated as the ratio of the effective dose at which 50% of the animals survived (ED_50_) of the standard curve, over the ED_50_ of the test curve.

### 5.7. Statistical Methods

For each MNA, probit analysis was used to fit a dose–response curve to the proportion of mice dead as a function of the base 10 logarithm of the antibody concentration. The NAC for each sample was estimated from the probit curves for the samples and associated reference standard. The ED_50_ of the associated standard was divided by the ED_50_ for the assay corresponding to the test sample.

For assays that failed due to excessive mortality, sample-specific lower limits of quantitation (LLOQs) were derived from hypothetical MNAs, in which the test curve just met the assay acceptance criteria and had the largest ED_50_ obtainable within the range of antibody concentrations tested. The observed standard curve ED_50_ (from the standard curve run concurrently with the test sample) was also incorporated, to allow for day-to-day variation in the performance of the assay. Thus, the LLOQ was calculated as the observed standard curve ED_50_ divided by the hypothetical test curve ED_50_. An adjusted NAC was then calculated with a value equal to one-half the LLOQ for the test curve.

The arithmetic mean was calculated for each BoNT serotype, dose route, and control or treatment group. To determine the average NAC per group, the sum of the individual NACs was divided by the total number of assays that passed the assay control acceptance criteria.

Linear regression and correlation analysis was performed using Prism 9.0 (GraphPad Software LLC, San Diego, CA). The data point at 0.0 was included in the linear regression. Pearson correlation and two-tailed *p* values were calculated using Prism 9.0.

## Figures and Tables

**Table 1:** Pharmacokinetic parameters from compartmental analysis of anti-BoNT/A mAbs.

mAb	Dose (mg/kg), Route	Model	C_0_ (μg/mL)	T_max_ (h)	β-Phase t_1/2_ (h)	t_1/2_ Abs (h)	t_1/2_ Elim (h)	AUC_inf_ (h*μg/mL)
XA-a	3, IV	2-compartment	20.3	0	365		201	5880
XA-b		2-compartment	9.83	0	5200		447	6330
XA-c		2-compartment	66.9	0	73.5		14.8	1430
XA-a	3, IM	1-compartment	11.2	55.4		14.3	202	4000
XA-b		1-compartment	5.97	29.9		10.2	64.1	781
XA-c		1-compartment	9.07	42.0		13.8	78.2	1480

C_0_—the concentration immediately upon injection assuming instantaneous distribution throughout the central compartment; t_1/2_—half life; abs—absorption, elim—elimination; AUC_inf_—area under the curve from time zero extrapolated to infinity; Tmax—time for antibody to reach maximal concentration.

**Table 2:** Pharmacokinetic parameters from noncompartmental analysis of anti-BoNT/A mAbs.

mAb	Dose (mg/kg), Route	C_max_ (μg/mL)	T_max_ (h)	t_1/2_ Elim (h)	AUC_last_ (h*μg/mL)	F	Simulated Concentration at 336 h Post-dose (g/mL)
XA-a	3, IV	18.9	6	35.2	2410		3880
XA-b		9.75	2	93.9	1020		-
XA-c		16.4	2	34.4	1390		569
XA-a	3, IM	11.3	72	24.5	2260	94%	4170
XA-b		5.76	48	73	627	83%	344
XA-c		9.36	48	30.4	1370	99%	1140

C_0_—the concentration immediately upon injection assuming instantaneous distribution throughout the central compartment; t_1/2_—half life; elim—elimination; AUC_last_—area under the curve from time zero to last measurable timepoint; T_max_—time for antibody to reach maximal concentration.

**Table 3:** Pharmacokinetic parameters from compartmental analysis of anti-BoNT/B mAbs.

Administration Route	mAb	Dose (mg/kg)	Model	C_0_ (μg/mL)	T_max_ (h)	β-Phase t_1/2_ (h)	t_1/2_ Abs (h)	t_1/2_ Elim (h)	AUC_inf_ (h*μg/mL)
IV	XB-a	3	2-compartment	20.7	0	144		77.1	2300
	XB-b		2-compartment	17.2	0	115		59.4	1470
	XB-c		2-compartment	20.7	0	133		81.1	2420
IM	XB-a	3	1-compartment	8.19	36.1		6.71	254	3310
	XB-b		1-compartment	6.16	31.0		5.59	237	2300
	XB-c		1-compartment	9.3	40.7		8.08	236	3570

C_0_—the concentration immediately upon injection, assuming instantaneous distribution throughout the central compartment; 1.5 mg total anti-BoNT/B mAbs administered t_1/2_—half life; elim—elimination.

**Table 4:** Pharmacokinetic parameters from non-compartmental analysis of anti-BoNT/B mAbs.

Administration Route	mAb	Dose (mg/kg)	C_max_ (μg/mL)	T_max_ (h)	t_1/2_ Elim (h)	AUC_last_ (h*μg/mL)	F (%)	Simulated C_336_ (μg/mL)	Simulated C_504_ (μg/mL)
IV	XB-a	3	18.2	2	69.5	2060			1040
	XB-b		15.1	2	49.6	1380			361
	XB-c		18.8	2	77.2	2220			1000
IM	XB-a	3	8.52	48	38.1	1670	81	4480	
	XB-b		6.33	48	26.5	1210	88	2760	
	XB-c		9.69	48	46.3	1890	85	4520	

C_336_; C_504_—concentration at 336 and 504 h post-dose, respectively; 1.5 mg total anti-BoNT/B mAbs were administered; C_max_—the maximal concentration; T_max_—time that maximal concentration is observed; t_1/2_ elim—elimination half-life; AUC_last_—area under the curve from time zero to last measurable timepoint; F—fraction absorbed.

**Table 5:** Parameters from linear regression and correlation of individual mAb concentration from ELISA or ECL vs. neutralizing antibody concentration (NAC).

	XA-a	XA-b	XA-c	All 3 Anti-A mAbs	XB-a	XB-b	XB-c	All 3 Anti-B mAbs
Best fit Slope (95% CI)	815.7 (614.0–1017)	453.8 (335.9–571.8)	618.9 461.4–776.5)	1888 (1428–2349)	324.8 (302.8–346.9)	253.6 (234–273.1)	358.3 (335.3–381.3)	880.3 (694.8–1066)
Goodness of fit	3855	2289	3011	8064	2169	1922	2261	6605
R^2^	0.6934	0.5765	0.6795	0.6949	0.8012	0.7659	0.8210	0.7436
*p* value	0.0028	0.0176	0.0036	0.0027	0.0005	0.0009	0.0003	0.0013

CI: Confidence interval. Linear regression and correlation calculated with Prism v9.0. The 0,0 data point included. Correlation was calculated using the average of replicates.

## Data Availability

We have not disclosed the sequences of the antibodies described here because their intended use is for biodefense. All other data is included in this manuscript.

## Abbreviations

ADA	Anti-drug antibody
AUC	Area under the curve
AUC_inf_	Area under the curve from zero timepoint extrapolated to infinity
AUC_last_	Area under the curve from zero timepoint to last measured timepoint
BAT^®^	Trade name of equine heptavalent Botulinum antitoxin
BIG	botulinum immune globulin
BoNT	Botulinum neurotoxin
BoNT/A	BoNT serotype A
BoNT/B	BoNT serotype B
BoNT/C	BoNT serotype C
BoNT/D	BoNT serotype D
BoNT/E	BoNT serotype E
BoNT/F	BoNT serotype F
C_0_	Concentration at zero timepoint (immediately after injection)
C_max_	Maximal concentration
CI	Confidence interval
ECL	Electrochemiluminescence
ED_50_	Effective dose at which 50% of the animals survived
ELISA	Enzyme-linked immunosorbent assay
F abs	Fraction absorbed (bioavailability)
FDA	Food and Drug Administration
ICCVAM	Interagency Coordinating Committee on the Validation of Alternative Methods
IM	Intramuscular
IP	Intraperitoneal
IV	Intravenous
LLOQ	Lower limit of quantitation
mAb	Monoclonal antibody
MNA	Mouse neutralization assay
MSD	Meso Scale Discovery
NAC	Neutralizing antibody concentration
NICEATM	National Toxicology Program Interagency Center for the Evaluation of Alternative Methods
NIH	National Institute of Health
PBS	Phosphate buffered saline
PK	Pharmacokinetic
QC	Quality control
t_½_ abs	Absorption rate half life
t_½_ elim	Elimination rate half-life
TMB	3,3′,5,5′-Tetramethylbenzidine
XA-a	monoclonal antibody that binds BoNT/A component of G03-52-01
XA-b	monoclonal antibody that binds BoNT/A component of G03-52-01
XA-c	monoclonal antibody that binds BoNT/A component of G03-52-01
XB-a	monoclonal antibody that binds BoNT/B component of G03-52-01
XB-b	monoclonal antibody that binds BoNT/B component of G03-52-01
XB-c	monoclonal antibody that binds BoNT/B component of G03-52-01
